# Acute prolonged motor aura resembling ischemic stroke after COVID − 19 vaccination (CoronaVac): the first case report

**DOI:** 10.1186/s10194-021-01311-w

**Published:** 2021-08-12

**Authors:** Wanakorn Rattanawong, Wasan Akaratanawat, Supatporn Tepmongkol, Aurauma Chutinet, Jarturon Tantivatana, Nijasri Charnnarong Suwanwela

**Affiliations:** 1 Division of Neurology, Department of Medicine, Faculty of Medicine, Chulalongkorn University, Bangkok, Thailand; 2 Department of Medicine, Faculty of Medicine, King Mongkut’s Institute of Technology Ladkrabang, Bangkok, Thailand; 3 Chulalongkorn Comprehensive Stroke Center, King Chulalongkorn Memorial Hospital, Bangkok, Thailand; 4 Chula Neuroscience Center, King Chulalongkorn Memorial Hospital, Bangkok, Thailand; 5 Division of Nuclear Medicine, Department of Radiology, Faculty of Medicine, Chulalongkorn University, Bangkok, Thailand; 6 Department of Radiology, Faculty of Medicine, Chulalongkorn University Biomedical Imaging Group (CUBIG), Chulalongkorn University, Bangkok, Thailand; 7 Department of Radiology, Faculty of Medicine, Chulalongkorn University, Bangkok, Thailand

**Keywords:** CoronaVac vaccine, Cortical spreading depression, Sinovac, Neurological deficit, COVID-19

## Abstract

**Background:**

We report the first case of a patient who suffered transient focal neurological deficit mimicking stroke following CoronaVac vaccination. However, instead of an ischemic stroke, motor aura was suspected.

**Case presentations:**

A 24 year-old Thai female presented with left hemiparesis fifteen minutes after receiving CoronaVac. She also had numbness of her left arm and legs, flashing lights, and headaches. On physical examination, her BMI was 32.8. Her vital signs were normal. She had moderate left hemiparesis (MRC grade III), numbness on her left face, arms, and legs. Her weakness continued for 5 days. A brain CT scan was done showing no evidence of acute infarction. Acute treatment with aspirin was given. MRI in conjunction with MRA was performed in which no restricted diffusion was seen. A SPECT was performed to evaluate the function of the brain showing significant hypoperfusion of the right hemisphere. The patient gradually improved and was discharged.

**Discussions:**

In this study, we present the first case of stroke mimic after CoronaVac vaccination. After negative imaging studies had been performed repeatedly, we reach a conclusion that stroke is unlikely to be the cause. Presumably, this phenomenon could possibly have abnormal functional imaging study. Therefore, we believed that it might be due to cortical spreading depression, like migraine aura, which we had conducted a literature review.

## Introduction

Due to the COVID-19 pandemic, Thailand has started its vaccination program since February 2021. The two approved vaccines are the CoronaVac (Covid-19 vaccine Vero cell, inactivated, manufactured by Sinovac Life Sciences, China) and the Oxford-Astrazenca®. After the launch of the mass vaccination, there were reports of patients who suffer unusual hemiparesis across the country, causing grave concern to individuals and medical personals. In this study, we report a case of a 24-year-old female who presented with acute neurological symptoms immediately after CoronaVac vaccination.

## Case report

**Fig. 1:**
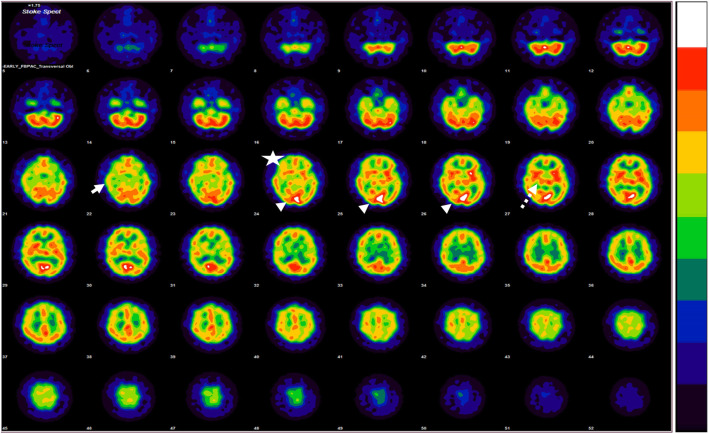
Axial single photon emission computer tomography with Tc-99 m ECD shows hypoperfusion to the right cerebral hemisphereinvolving right inferior frontal (star),temporal (white arrow), occipital regions (arrow head) and right thalamus (dashed arrow)

A 24-year-old Thai female nurse aide was referred to King Chulalongkorn Memorial Hospital due to acute left hemiparesis after receiving the first dose of CoronaVac. Fifteen minutes after the injection at the left deltoid, she developed visual disturbance characterized by bright flashing lights in both eyes that lasted 5 min, followed by marked nausea and vomiting. She was then transferred to the emergency department of a provincial hospital. Twenty minutes later, she noticed a tingling sensation over the fingers in her left hand, followed by numbness and weakness of the left arm. Brain computed tomography (CT) was immediately performed and results showed no evidence of infarction. Upon coming out of the CT scanner, she started to feel that her left leg was also weak and numb. A magnetic resonance imaging (MRI) was done and showed no abnormalities. Given the uncertainty of the cause of the symptom, thrombolytic therapy was not given. Instead, aspirin was initiated. On the next day, she reported a “migraine-like” pulsatile headache at her left temporal area, radiated to the neck for 2 h and then disappeared after oral ibuprofen.Her weakness and numbness remained the same for two days. No fever had been presented during her admission; accordingly, she was then transferred to King Chulalongkorn Memorial Hospital for further investigations. From the examination, she was a young female with a BMI of 32.8. Her blood pressure was 120/70 mmHg. She still had a moderate degree of left hemiparesis involving the arm and leg (MRC III). The numbness was dense over her left arm and left side of her face, while her left leg had a lesser degree of decreased pinprick sensation. Her left arm tone was decreased. Proprioception and vibration senses were intact. Her past medical history included menstrual-related migraines without aura which occurs 2–3 times per year. She denied a family history of migraine and hemiplegic migraine. Despite the persistent weakness for 3 days, the follow-up brain MRI showed no abnormalities. On MRA, mild irregularity of the left pericallosal artery was suspected, which may indicate vasospasm. Oral nimodipine was initiated. Since the unusual presentation cannot be explained by structural imaging, single-photon emission computed tomography (SPECT) using Tc-99 m ethylcysteinate dimer (ECD) was performed. Results demonstrated relative hypoperfusion to the right cerebral hemisphere involving the right inferior frontal, temporal, occipital regions, and right thalamus (Fig. 1). Whole exome sequencing studies were negative for hemiplegic migraine genes (CACNA1A, ATP1A2, and SCN1A). But, variants on three genes were identified (CD38, GPNMB, and MYH7). Two days after admission (5 days after vaccination and symptom onset), the numbness and weakness gradually resolved.

## Discussion

We report a case of a young female patient with no known family history of hemiplegic migraine who presented with transient neurological deficits immediately after CoronaVac immunization. The acuteness of the left hemiparesis pattern may resemble ischemic stroke causing controversy over the treatment. Despite the existing neurological deficit, the normal MRI findings on 2 separate occasions suggest that the diagnosis of ischemic stroke was unlikely. On the contrary, we believe that the positive visual disturbance and progressing unilateral numbness and weakness for more than 20 min suggest the pathophysiology of a migraine aura, which is likely to be a part of hemiplegic migraine. In addition, migraine-like headaches during the course and the complete resolution of all symptoms without evidence of structural brain abnormalities strongly support the hypothesis. Even though the patient’s headache pattern did not meet the criteria for The International Classification of Headache Disorder third edition (ICHD III) [1], migraine headache was still the principal diagnosis since the duration of headache could be altered by the treatment of ibuprofen. Although the patient had menstrual related migraines without aura as her underlying disease, the family history of hemiplegic migraine and gene analysis were all negative. Therefore, the first attack of sporadic hemiplegic migraine might be suspected. Furthermore, variant genes were identified but none had relationship with familial hemiplegic migraine.

Regarding the SPECT, the asymmetrical abnormalities with relative hypoperfusion to the right cerebral hemisphere found on SPECT imaging could explain the ongoing neurological deficit. We believe that the hypoperfusion on the occipital lobe could explain the abnormal flashing lights, while the sensory abnormality could be explained by the relative hypoperfusion of the thalamus and her persistent motor weakness could be originated from the motor region. Compared with previous reports, prolonged aura symptoms in hemiplegic migraine could have relative hypoperfusion or hyperperfusion on the affected brain hemisphere depending on the timing of the imaging [2, 3]. In case of acute prolonged auras, it usually demonstrates a hypoperfusion pattern. However, the exact timing is unknown. Unlike visual auras, motor auras show multifocal hypoperfusion rather than a posterio-anterior propagation of hypoperfusion. All of this evidence support all the phenomenon in our case. Therefore, it is believed to be a prolonged motor aura.

Cortical spreading depression (CSD) has long been described to be the pathophysiology of migraine aura [4]. The mechanism is thought to be a transient wave of depolarized neurons propagating the cerebral cortex at a rate of 2–5 mm/min, and therefore causing transient focal neurological deficit. It is still under debate whether CSD triggers migraine headaches or it is simply just a co-phenomenon. Motor aura [5], a rare aura type, is found in both familial and sporadic hemiplegic migraines. It is characterized as a transient unilateral (rarely bilateral) motor weakness usually preceding migraine headaches but could also occur with the headache or even when the headache has subsided [6]. Normally, motor aura endures between 20 and 60 min. Interestingly, it could last for several days or even weeks. In addition to motor aura, other aura types including visual, sensory, speech and/or language, brainstem, or retinal auras could concomitantly occur in hemiplegic migraine headaches [7]. Despite its prolonged aura duration, most of the cases fully recover with no permanent neurological damages, but some cases have reported infarction on MRI [8]. The pathogenesis explaining prolonged weakness is unknown but is believed to be due to reverberating spreading depression wave [9]. This phenomenon causes a release in glutamate and thereafter activating the N-methyl-D-aspartate (NMDA) receptors. Therefore, causing persistent ongoing weakness [10]. Another interesting fact about CSD is that it is easily triggered in females. In animal studies, female mice have a lower induction threshold of CSD in which sex hormone might play a crucial role [11]. Regarding these facts and carefully review our patient history, we, therefore, presume that the vaccine injection might be the crucial element to trigger CSD.

Regarding the pathophysiology of the CSD, it is well documented that migraine could be easily exacerbated in vulnerable individuals especially during the perimenstrual period [12] like in our case. It is believed that sex hormone plays a crucial role in the trigger. Nevertheless, there are more cases of focal neurological deficits after CoronaVac vaccination in Thailand that are currently under investigation and have not been reported. We believed that the cause of the symptoms is due to the vaccine per se. Unlike other COVID-19 vaccines, CoronaVac [13] is composed of inactivated SARS-CoV 2 virus (grew in Vero cells and deactivated with β-propiolactone) and aluminum hydroxide as an adjuvant to enhance immunity. After carefully reviewing the composition of the vaccine, we doubt that aluminum might be responsible for the reverberating spreading depression wave. It is believed that aluminum is responsible for the disruption of the Glutamate – Nitric oxide – cGMP pathway in a study using rat model, resulting in the release of the nitric oxide and activation of the nitric oxide system [14]. Later, nitric oxide triggers the production of glutamate [15] and consequently activates the NMDA receptors causing the prolonged aura. Yet, the toxic level is unclear and has not been studied. In our case, we believed that nitric oxide plays a crucial role in inducing migraine aura and headache [16]. It should be noted that it is also responsible for the prolongation of the aura later in the course of the disease. However, this theoretical postulation has yet to be investigated.

In conclusion, we report a patient with neurological deficits after COVID-19 vaccination and propose CSD as the mechanism of these transient symptoms.

## Data Availability

Data is available with the corresponding author upon request.
